# Intrinsic Kidney Pathology in Children and Adolescents Following COVID-19 Vaccination: A Systematic Review

**DOI:** 10.3390/children9101467

**Published:** 2022-09-26

**Authors:** Henry H. L. Wu, Mohan Shenoy, Philip A. Kalra, Rajkumar Chinnadurai

**Affiliations:** 1 Renal Research Laboratory, Kolling Institute of Medical Research, Royal North Shore Hospital, St. Leonards, Sydney, NSW 2065, Australia; 2 Department of Pediatric Nephrology, Royal Manchester Children’s Hospital, Manchester University NHS Foundation Trust, Manchester M13 9WL, UK; 3 Faculty of Biology, Medicine and Health, University of Manchester, Manchester M13 9PG, UK; 4 Department of Renal Medicine, Salford Royal Hospital, Northern Care Alliance Foundation Trust, Salford M6 8HD, UK

**Keywords:** intrinsic kidney pathology, children, adolescents, COVID-19 vaccination, systematic review

## Abstract

Global COVID-19 vaccination programs for children and adolescents have been developed with international clinical trial data confirming COVID-19 mRNA vaccine safety and efficacy for the pediatric population. The impact of COVID-19 vaccination in the kidneys is thought to be explained by a complex immune-mediated relationship between the two, although the pathophysiological mechanisms of how COVID-19 vaccination potentially induces kidney pathology are not presently well known. Whilst intrinsic kidney pathologies following COVID-19 vaccination have been reported in adults, such cases are only being recently reported with greater frequency in children and adolescents. Conforming to the PRISMA checklist, we conducted a systematic review of the current literature to provide an overview on the range of intrinsic kidney pathologies that have been reported following COVID-19 vaccination in children and adolescents. All English language research articles published on or before 30 June 2022 reporting new-onset or relapsed intrinsic kidney pathology in children or adolescents (≤18 years) following COVID-19 vaccination were selected for qualitative analysis. Out of 18 cases from the 13 published articles selected, there were 10 cases of IgA nephropathy (1 case of rapidly progressive glomerulonephritis requiring acute hemodialysis), 5 cases of minimal change disease (MCD), 1 case of concurrent MCD/tubulointerstitial nephritis (TIN) and 2 cases of TIN. There is no indication currently to avoid vaccination, unless specific circumstances exist, as the benefits of COVID-19 vaccination far outweigh its risks. Concluding the findings from our systematic review based on preliminary evidence, potential adverse effects to the kidney from COVID-19 vaccination affects a small number of children and adolescents among the many who have been vaccinated. There remains good reason at present to support vaccination of children and adolescents with a greater morbidity status, such as those living with preexisting chronic kidney disease. Close observation of all children and adolescents receiving COVID-19 vaccination is recommended, particularly in those with preceding intrinsic kidney pathology to identify risks of relapsed disease.

## 1. Introduction

The Coronavirus disease 2019 (COVID-19) pandemic has enormous effects globally, with the disease capable of leading to greater risk of morbidity and mortality within communities [1,2]. There were 6.45 million deaths which occurred worldwide amongst individuals identified with acute COVID-19 infection by the end of July 2022 according to World Health Organization registry data, whether these were directly or indirectly caused by COVID-19 infection [1]. At present, there remain no definitive treatments or prevention strategy to reduce COVID-19 manifestations and mortality. Mass vaccination programs have emerged as the prime strategy from governments to reduce COVID-19 prevalence, following the innovations of various vaccinations targeting COVID-19 [3]. The introduction of vaccination programs complement public health infection mitigation measures that are already advocated since early days of the pandemic [3]. Whilst COVID-19 vaccination programs for replication-defective viral-vectored and messenger RNA (mRNA) vaccines have been ongoing for adults over the past 2 years, administering COVID-19 mRNA vaccinations (Pfizer-BioNTech BNT162b2, Moderna mRNA-1273 from June 2022) for children and adolescents was only recently approved and recommended to be administered through specific regimes only, as clinical trial data on COVID-19 mRNA vaccine safety and efficacy for children and adolescents emerged with greater clarity [4,5,6,7]. All of the clinical trials evaluating safety of the Pfizer-BioNTech BNT162b2 and Moderna mRNA-1273 vaccines have not identified any cases of severe adverse effects in any of the study patients [4,5,6]. The mechanism of mRNA vaccines involve lipid nanoparticle nucleoside-modified mRNA encoding the severe acute respiratory syndrome–related coronavirus-2 (SARS-CoV-2) spike protein, mediating host attachment and SARS-CoV-2 viral entry [8,9].

The effects of COVID-19 vaccination on the kidneys have been widely discussed. It is presumed that a complex immune-mediated relationship exists between the development of various kidney histopathologies and COVID-19 vaccination, although the responsible pathophysiological mechanisms are not well-established as of yet [10,11]. Data relating to the epidemiology, pathophysiology, risk factors and prognosis of individuals with new-onset and relapsed intrinsic kidney pathology following COVID-19 vaccination is reported with greater frequency in adults compared to children and adolescents [12,13,14,15]. Gradually, case reports have been published describing new-onset or relapsed cases of podocytopathy, glomerular disease and other intrinsic kidney pathologies in children and adolescents following COVID-19 vaccination, given that greater numbers of children and adolescents have been receiving COVID-19 vaccinations worldwide. Most of the reported cases are histologically diagnosed following kidney biopsy, whereas some are empirical diagnoses determined through medical history relating to kidney disease and correlation with time of COVID-19 vaccination, non-invasive investigations, and treatment response. Determining the mechanisms of how intrinsic kidney pathology manifests in children and adolescents following COVID-19 vaccination could be more challenging compared to adults, due to the ethical limitations of pursuing invasive kidney biopsy for young children, and the lack of a reliable non-invasive diagnostic test at present [16].

There have been no systematic reviews published to summarize the findings of intrinsic kidney pathology following COVID-19 vaccination in children and adolescents as of July 2022. We conducted a systematic clinical review of the current literature to delineate the range of intrinsic kidney pathologies that have manifested following COVID-19 vaccination in children and adolescents.

## 2. Materials and Methods

### 2.1. Eligibility Criteria

All research articles reporting new-onset or relapsed intrinsic kidney pathology in children or adolescents (≤18 years) following COVID-19 vaccination were included. Articles for inclusion described intrinsic kidney pathology in both native and transplanted kidneys. We only selected full-text articles published in the English language for review. Only studies published on or before 30 June 2022 were included.

### 2.2. Search Strategy and Study Selection

**Figure 1:**
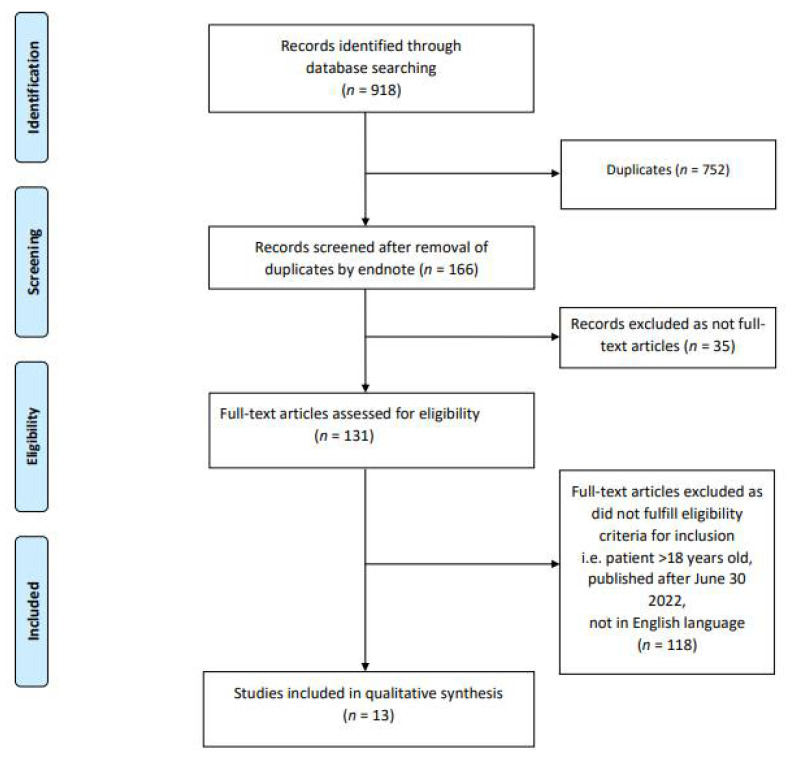
PRISMA Flow Diagram.

A systematic literature search was conducted by two independent authors (H.H.L.W. and R.C.) in the following databases: “PubMed”, “Web of Science”, “EMBASE” and “Medline-ProQuest”. The search terms incorporated the following: “COVID-19 Vaccination” AND “Kidney Histopathology” AND “Children”; “SARS-CoV-2 Vaccination” AND “Kidney Histopathology” AND “Children”; “COVID-19 Vaccination” AND “Renal Histopathology” AND “Children”; “SARS-CoV-2 Vaccination” AND “Renal Histopathology” AND “Children”; “COVID-19 Vaccination” AND “Kidney Manifestations” AND “Children”; “COVID-19 Vaccination” AND “Renal Manifestations” AND “Children”; “SARS-CoV-2 Vaccination” AND “Kidney Manifestations” AND “Children”; “SARS-CoV-2 Vaccination” AND “Renal Manifestations” AND “Children”; “COVID-19 Vaccination” AND “Nephrotic Syndrome” AND “Children”; “SARS-CoV-2 Vaccination” AND “Nephrotic Syndrome” AND “Children”; “COVID-19 Vaccination” AND “Glomerulonephritis” AND “Children”; “SARS-CoV-2 Vaccination” AND “Glomerulonephritis” AND “Children”; “COVID-19 Vaccination” AND “Nephropathy” AND “Children”; “SARS-CoV-2 Vaccination” AND “Nephropathy” AND “Children”; “COVID-19 Vaccination” AND “Kidney Histopathology” AND “Adolescents”; “SARS-CoV-2 Vaccination” AND “Kidney Histopathology” AND “Adolescents”; “COVID-19 Vaccination” AND “Renal Histopathology” AND “Adolescents”; “SARS-CoV-2 Vaccination” AND “Renal Histopathology” AND “Adolescents”; “COVID-19 Vaccination” AND “Kidney Manifestations” AND “Adolescents”; “COVID-19 Vaccination” AND “Renal Manifestations” AND “Adolescents”; “SARS-CoV-2 Vaccination” AND “Kidney Manifestations” AND “Adolescents”; “SARS-CoV2 Vaccination” AND “Renal Manifestations” AND “Adolescents”; “COVID-19 Vaccination” AND “Nephrotic Syndrome” AND “Adolescents”; “SARS-CoV-2 Vaccination” AND “Nephrotic Syndrome” AND “Adolescents”; “COVID-19 Vaccination” AND “Nephropathy” AND “Adolescents”; “SARS-CoV-2 Vaccination” AND “Nephropathy” AND “Adolescents”; “COVID-19 Vaccination” AND “Glomerulonephritis” AND “Adolescents”; “SARS-CoV-2 Vaccination” AND “Glomerulonephritis” AND “Adolescents”. The articles were screened by H.H.L.W. and R.C. for relevance and duplicate publications were removed. Duplicate screening and the eligibility check was performed by both H.H.L.W. and R.C. The study selection process was carried out using the Preferred Reporting Items for Systematic Reviews and Meta Analyses (PRISMA) guidelines (Figure 1).

### 2.3. Data Extraction

If available, data including patient demographics (age, sex and ethnicity), co-morbidities, time to presentation from the day of previous vaccination, clinical presentation, brand of vaccine administered, number of vaccine doses given, kidney parameters pre-vaccination and throughout the time period of acute presentation until most recently reported follow-up (serum creatinine, serum albumin, presence and degree of proteinuria and hematuria), information on whether kidney biopsy was performed, treatment received following diagnosis and clinical outcome following treatment, were extracted from the included articles. These data are described in the results section of this article, and also presented in tabular form.

### 2.4. Study Registration

A pre-defined review protocol was registered at the PROSPERO international prospective registry of systematic reviews, under registration number CRD42022331838.

## 3. Results

Our systematic literature search selected 13 articles describing a total of 18 cases of new-onset or relapsed intrinsic kidney pathologies following COVID-19 vaccination in children and adolescents. None of the included cases have had COVID-19 infection diagnosed prior to vaccination. Of these 18 cases, the predominant pathology was IgA nephropathy (10 cases) (Table 1) followed by nephrotic syndrome (6 cases) and tubulointerstitial nephritis (TIN) (2 cases) (Table 2).

### 3.1. IgA Nephropathy

The mean age of the 10 cases reported with IgA nephropathy was 15 years with a 1:1 male: female ratio. The median time between the day of vaccination and clinical presentation was 1 day. Seven of the ten cases were considered of new-onset with the other three being relapses of histologically diagnosed IgA nephropathy. Amongst the seven new-onset IgA nephropathy cases, four patients have been followed up in clinic for asymptomatic hematuria previously, with no formal histological diagnosis of IgA nephropathy or other intrinsic kidney pathologies. Most of the reported cases occurred following the Pfizer BNT162b2 vaccine (8 cases) and the vaccine brand was unknown in two cases. The majority of patients presented following the second dose of COVID-19 vaccination (7 cases). All of the reported cases presented with gross macroscopic hematuria with varying degrees of proteinuria, of which there is one case of rapidly progressive glomerulonephritis requiring acute hemodialysis because of oliguria. The predominant histopathological changes included crescentic changes in light microscopy and diffuse mesangial deposits of IgA and C3 in immunofluorescence. All patients who had crescentic glomerulonephritis received intravenous pulsed methylprednisolone followed by oral prednisolone and the rest received supportive therapy. Resolution and recovery of kidney function was noted in all cases following treatment [17,18,19,20,21,22,23].

### 3.2. Nephrotic Syndrome and Tubulointerstitial Nephritis

Nephrotic syndrome was the second commonest clinical presentation to be reported (6 cases) with four being new-onset and two cases being relapses. The mean age of the reported cases was 16 years with the majority of patients being males (4 cases) and having followed Pfizer BNT162b2 vaccine (4 cases). The median time between the day of vaccination and clinical presentation was 7 days. For patients who had kidney biopsies, histopathological changes were predominantly typical of minimal change disease (MCD). One patient presented with an overlap of MCD and TIN. All reported cases had good response to steroid treatment [24,25,26,27,28].

There were two patients presenting with TIN following Pfizer BNT162b2 vaccination. Whist one received steroid treatment, the other case recovered with supportive treatment [29].

## 4. Discussion

IgA nephropathy appeared as the most frequently reported intrinsic kidney pathology following COVID-19 vaccination. IgA nephropathy is the most common glomerular disease in children and adolescents, with incidences of up to 30% in countries such as Japan and Korea although the global incidence remains variable [30,31]. Approximately 30% of children and adolescents with IgA nephropathy will develop kidney failure within 20 years from diagnosis [32]. The current body of evidence suggests that IgA nephropathy is an immune complex-mediated disease whose pathophysiology involves three immunological processes-excessive production of galactose deficient IgA1 by gut lymphocytes, followed by development of IgG autoantibodies against galactose-deficient IgA1, and mesangial deposition of immune complexes in the kidney superadded by the dysregulation of soluble CD89 (CD89 is an Fc receptor for IgA) and transglutaminase 2 [33,34]. These immunological processes may be induced by an individual’s genetic predisposition (i.e., gene variants encoding galactosylation) and various environmental factors (i.e., infections, dietary imbalances) [33,35]. Vaccine-induced IgA nephropathy was reported in the context of influenza vaccinations, which is annually provided in health systems across most developed countries [36]. It was observed that intramuscular inactivated influenza vaccines elicited hyperresponsiveness in patients with existing IgA nephropathy, leading to excess production of IgA1 monomers [37].

New-onset and relapsed cases of IgA nephropathy have been increasingly reported in adults following COVID-19 vaccination. A greater level of anti-glycan antibody production may partly explain the association between COVID-19 vaccination and IgA nephropathy, given that anti-glycan antibodies cross-react with poorly galactosylated IgA1 and that mucosal immune responses are not stimulated following COVID-19 vaccination [12,38,39]. For mRNA vaccines, another explanation for the association between COVID-19 vaccination and IgA nephropathy relates to increased antibody production [12], as this type of vaccine induces a more potent T-helper cell and B-cell response in the germinal center. It is also acknowledged that many healthy individuals develop spiked IgA production following mRNA-based vaccinations, although the mechanism for this remains unclear [40].

Another scenario which might explain the apparent association between COVID-19 vaccination and IgA nephropathy is the uncovering of subclinical IgA nephropathy following COVID-19 vaccination with the onset of gross hematuria. In Japan, where the majority of IgA nephropathy cases were reported in children and adolescents following COVID-19 vaccination, it was noted that most of these patients (previously with asymptomatic hematuria) were only formally diagnosed as IgA nephropathy because of post-vaccination gross hematuria [19]. Otherwise, they would have continued follow-up in pediatric nephrology clinics once or twice per year for review of asymptomatic hematuria without active intervention as per guidance from the Japan Society of School Health and the Japanese Society for Pediatric Nephrology guidelines [19,41]. The updated Japanese Society for Pediatric Nephrology guidelines have detailed firm guidance for follow-up of asymptomatic hematuria, with kidney biopsy in scenarios where there is persistent proteinuria or hematuria, and recurrent gross hematuria [41]. These measures allow for timely diagnosis of intrinsic kidney pathologies in children and adolescents, whether this is in the context of COVID-19 vaccinations or not.

Nevertheless, detailed evaluation of the 10 IgA nephropathy cases following COVID-19 vaccination in our systematic review did not identify any significant trends between pre-vaccination tests and the timing of onset of post-vaccination symptoms relating to IgA nephropathy. There appear no clear causative associations between COVID-19 vaccination and onset of IgA nephropathy at present. The lack of published literature describing COVID-19 vaccination associated IgA nephropathy is a major limitation, and perhaps more accumulated cases leading to a greater sample size of this presentation would further clarify these associations.

Kidney pathology associated with the clinical presentation of nephrotic syndrome has been reported in children and adolescents following COVID-19 vaccination. MCD encompasses more than 80% of the cases of nephrotic syndrome presentations in children and adolescents [42,43]. The histological changes which occur with the MCD disease process involves the loss of processes at the glomerular visceral epithelial cell-foot, resulting in podocyte effacement, vacuolation and a more rapid growth of microvilli structures in the visceral epithelial cells [43,44,45]. Therefore, patients with MCD are more susceptible to excess proteinuria. The overwhelming majority of nephrotic syndrome presentations in children and adolescents are idiopathic (95% of cases), with the remaining (5% of cases) likely secondary to causes ranging from viral [e.g., Hepatitis B and C, Human Immunodeficiency Virus (HIV)] and inflammatory diseases (e.g., Multisystem inflammatory syndrome in children, Juvenile Idiopathic Arthritis) to less common conditions such as Amyloidosis and Henoch-Schonlein Purpura [46,47,48,49,50,51]. Prior to the COVID-19 pandemic, onset of MCD secondary to vaccination has been reported following the administration of influenza, hepatitis B, pneumococcal, and measles to tetanus–diphtheria–poliomyelitis vaccines [52,53,54,55]. The current theory of MCD pathogenesis is that the condition manifests from complex interactions between T-cells, B-cells, circulating factors, and podocytes [56]. Dysregulation of T-cell mediated immunity is widely speculated to be the main cause of COVID-19 vaccine-induced MCD, with enhanced type 2 T-helper cell activity causing release of cytokines such as interferon-γ and IL-2 leading to increased permeability factor formation [12,57]. This is in contrary to the antibody mediated immune response mechanism purported in COVID-19 vaccine-induced IgA nephropathy. The time for symptom onset in MCD is longer for most reported cases compared to IgA nephropathy.

The three histologically diagnosed cases of TIN in children and adolescents following COVID-19 vaccination are novel. There was a lack of clear-cut co-morbidities and risk factors which might have provided non-COVID-19 explanations for this pathology, but TIN is often an underdiagnosed and under-reported condition and causality is difficult to prove given a myriad of potential confounding factors [58]. The attention directed towards TIN is less compared to glomerular diseases and there are many unanswered questions regarding the temporal association between COVID-19 vaccination and TIN [59,60].

There was another novel case of antineutrophil cytoplasmic antibody (ANCA)-negative and anti-glomerular basement membrane (GBM)-negative crescentic glomerulonephritis manifesting in a 16-year old girl, who presented with dyspnea and headache approximately 6 weeks after receiving her second dose of the Pfizer BioNTech BNT162b2 vaccine [61]. The patient required acute dialysis for 2 weeks, and subsequently recovered with oral steroid and mycophenolate mofetil therapy. This case report by Kim and colleagues was published in July 2022, and hence not included in our systematic review. The authors postulated that vaccine-induced autoimmunity may have caused the development of subsequent glomerulonephritis after mRNA-based vaccination, similar to that of the IgA nephropathy cases.

From the 18 cases reported in our systematic review, the majority of cases were reported in Asia (12 cases) in which Japan (7 cases, with 6 of the 7 cases being IgA nephropathy) as most prominent. This could largely be attributed to the well-established national school urinalysis screening system in Japan, where successes in early detection of subclinical glomerulonephritis have been documented [41,62,63]. Furthermore, it is noticeable that all of the case reports occurred in developed countries (all in the ‘very high’ category of the inequality-adjusted human development index) [64]. mRNA vaccination programs for children and adolescents were only introduced in developed countries in recent months, with pediatric-dosed vaccines now being more available and accessible. No international registry data is currently available documenting the number of vaccine doses administered to children and adolescents throughout the time period covered by our systematic review. Individual national registries such as those from the United States (e.g., American Academy of Pediatrics) and Australia (e.g., Australian Government Department of Health and Aged Care) have recorded the prevalence of children and adolescents receiving COVID-19 vaccinations during this period [65,66]. For example, between March 11 2022 and 29 June 2022 in the United States, 36% of children aged 5 to 11 received the 1st dose whilst 28% of children received the 2nd dose of either Pfizer-BioNTech BNT162b2 or Moderna mRNA-1273 vaccine [65]. During a similar time period in Australia for children aged 5 to 15 years, 52.3% received the 1st dose whilst 39.5% of children received the 2nd dose of either Pfizer-BioNTech BNT162b2 or Moderna mRNA-1273 vaccine [66]. Due to the recency of COVID-19 vaccine roll-out for children and adolescents, our understanding of any indication of a global rise in kidney injury incidence since the period of vaccine roll-out for children and adolescents is limited at present. Going forward, we anticipate efforts to establish international registry data to monitor for this.

Overall, the relative lack of reported cases at present makes it difficult to synthesize a summary of overall findings and be confident about the links, if any, between the number of COVID-19 vaccination doses and risks of developing intrinsic kidney pathology. There are no case–control or cohort studies available in the topic area to conduct a meta-analysis. Hence, the article is presented as a descriptive systematic review with only case reports being identified from our search process. Furthermore, our analysis was inevitably limited in concluding significant associations between baseline morbidity profile, investigation results (e.g., serum creatinine), the nature of one’s acute clinical presentation and their eventual outcomes amongst the included cases. Exact duration of hospitalization was only documented for 2 cases amongst those included for detailed evaluation, and the completeness and detail in which serum and urinary investigation results were reported by authors of the included cases have been inconsistent. The majority of these cases occurred too recently for us to determine how patients have evolutionized post-treatment from the reported episode of intrinsic kidney pathology developing following COVID-19 vaccination. Apart from the single case of IgA nephropathy with rapidly progressive glomerulonephritis in which acute hemodialysis was indicated because of oliguric symptoms, all of the other cases included in this systematic review recovered well from their presenting episode with supportive treatment and/or steroid therapy. It is also quite possible that children presenting with IgA nephritis following vaccination actually had previously undiagnosed IgA disease.

## 5. Conclusions

In conclusion, our systematic review summarizes currently reported intrinsic kidney pathologies which manifested in children and adolescents following COVID-19 vaccination. Based on these preliminarily reported cases, the potential immune-mediated relationship between COVID-19 vaccines and onset of various intrinsic kidney pathologies has been explored. It should be acknowledged that the number of reported cases of intrinsic kidney pathology developing following COVID-19 vaccination is very small in relation to the hundreds of millions of vaccinations that have already occurred in children and adolescents, and that the protective benefits offered by COVID-19 vaccinations far outweigh its risks. At present, there is no indication to avoid vaccination unless specific circumstances exist. On the contrary, there have been reported serious complications associated with COVID-19 infection in pediatric patients with kidney disease, which reinforces the importance of COVID-19 vaccination in this vulnerable group [67]. In the future, we recommend the continued development and expansion of COVID-19 vaccination programs worldwide and keeping a watchful eye on children and adolescents with preexisting kidney disease. From the recency of cases included in this systematic review, and the relative lack of published data addressing the association between development of new-onset or relapsed intrinsic kidney pathologies and COVID-19 vaccination in children and adolescents, further reports and primary research will be needed to provide greater clarification on possible pathophysiological associations.

## Figures and Tables

**Table 1:** Demographics and outcomes of children and adolescents with new-onset and relapsed IgA nephropathy following COVID-19 vaccination.

Author(s) & Country of Report	Age (yrs)	Sex	Time to Presentation from Day of Vaccination (Days)	Comorbidities	New- onset or Relapse	Vaccine Brand & Vaccine Dose	Serum Creatinine Since Day of Presentation (mg/dL)	Serum Albumin Since Day of Presentation (g/dL)	Urine Protein-to-Creatinine Ratio Since Day of Presentation (g/g)	Haematuria Since Day of Presentation	Kidney Biopsy	Treatment Received	Clinical Outcome
Hanna et al. [17], United States	13	M	Within 1 day	TIDM, previous diagnosis of IgA nephropathy	Relapse	Pfizer, 2nd dose	Pre-vaccination: 0.54 Day 2: 1.31 Day 6: 0.66	Pre-vaccination: 3.4 Day 2: 3.8 Day 6: 3.0	Pre-vaccination: 1.6 Day 2: 1.07 Day 6: 0.86	Day 1: Gross haematuria, resolved after 2 days	Not performed	Supportive treatment. Lisinopril 10 mg/day commenced for 5 days.	IgA nephropathy self-resolved within 1 week without other interventions
Hanna et al. [17], United States	17	M	Within 1 day	Nil	New-onset	Pfizer, 2nd dose	Day 6: 1.78 Day 9: 1.47 Day 22: 1.20	Day 9: 3.8	Day 9: 1.75	Day 1: Gross haematuria, resolved after 4 days	Cellular glomerular crescents and moderate to severe tubulointerstitial scarring suggestive of pre-existing IgA nephropathy	IV pulsed methylprednisolone 1 g daily and oral prednisolone	Improved serum creatinine on follow-up following steroid treatment
Horino et al. [18], Japan	17	M	2 days	Nil. But urine dipstick 5 months ago revealed microscopic haematuria	New-onset	Pfizer, 2nd dose	Day 1: 0.70	Not specified	Day 1: 1.00 Day 7: 1.40	Day 1: Gross haematuria, >100 RBC/HPF Day 7: Gross haematuria, >100 RBC/HPF	Light microscopy revealed mesangial cell and matrix proliferation, endocapillary hypercellularity and crescents. Immunofluorescence staining revealed predominantly IgA and C3 deposits with weak IgG deposits in the mesangial areas.	Initially treatment was supportive, but with persistence of proteinuria and microhaematuria after 2 months, tonsillectomy and pulsed steroid therapy was commenced	Persistence of proteinuria and microhaematuria after 2 months of supportive treatment. Patient referred for tonsillectomy and commenced on pulsed steroid therapy
Okada et al. [19], Japan	17	F	4 days	No cause found for microscopic haematuria when 7 yrs old, followed up in clinic	New-onset	Pfizer, 1st dose	Day 1: 0.58	Not specified	Day 1: 0.37 Day 10: 0.05 Day 16: 0.07	4 months pre-vaccination: 20–29 RBC/HPF 4 days after 1st dose: Gross haematuria	Light microscopy revealed mild mesangial proliferation, no endo-capillary or extra-capillary proliferation, sclerosis, or adhesion was observed. Immunofluorescence analysis revealed diffuse mesangial IgA and C3 deposits. Electron microscopy revealed electron-dense deposits in the mesangial lesions.	Supportive treatment. Patient proceeded to receive 2nd dose of Pfizer vaccine shortly after episode following 1st dose	Spontaneous resolving of proteinuria and gross haematuria, patient followed up until 16 days after 2nd dose of vaccine.
Niel et al. [20], Luxembourg	13	F	Within 1 day	Nil	New-onset	Pfizer, 1st dose	Day 1: 3.57 Day 11: 1.90 Day 30: 0.86	Not specified	Day 1: 3.88	Day 1: Macroscopic haematuria Day 30: Microscopic haematuria persisted	Mesangial and endocapillary proliferation. No constituted crescents were observed, but fibrin deposits were present in the Bowman space of most of the observed glomeruli. No evidence of renal scarring from previous kidney injury could be detected. Diffuse mesangial IgA and C3 deposits on immunofluorescence. Diffuse mesangial deposits on electron microscopy.	Patient commenced on haemodialysis in hospital as kidney function rapidly deteriorated and patient became oliguric. Patient also commenced on three IV methylprednisolone doses followed by oral prednisolone.	Improvements in kidney function following commencement of haemodialysis and steroid course. By 30 days post-vaccination, serum creatinine returned to normal range. Microscopic haematuria and slight proteinuria persisted
Morisawa et al. [21], Japan	16	M	Within the same day	Followed up in clinic for asymptomatic haematuria.	New-onset	Brand not noted, 2nd dose	Day 6: 1.1 Day 20: 1.26 Day 55: 1.29 Day 90: 1.05	Not specified	Day 6: 0.28	Day 1: Gross haematuria Day 3: Gross haematuria resolved	Mild proliferation of mesangial cells and cellular crescents was diagnosed in light microscopy.	Gross haematuria resolved 3 days post-vaccination. However, a rise in serum creatinine and proteinuria is observed afterwards. Patient was commenced on IV pulsed methylprednisolone for 3 consecutive days over two consecutive weeks. Oral prednisolone also prescribed and its dose increased with initial inability to improve serum creatinine.	At follow-up 3 months post-vaccination, significant improvement in serum creatinine has been observed.
Morisawa et al. [21], Japan	13	F	Within 1 day	Followed up in clinic for asymptomatic haematuria.	New-onset	Brand not noted, 2nd dose	Not specified	Not specified	Day 7: 1.99	2 months pre-vaccination: 10–20 RBC/HPF Day 1: Gross haematuria Gross haematuria gradually resolved	Light microscopy found histological picture of IgA nephropathy with mild proliferation of mesangial cells	Supportive treatment	Proteinuria resolved on day 26 post-vaccination spontaneously
Udagawa et al. [22], Japan	15	F	1 day	IgA nephropathy. Patient was in remission with multiple-drug therapy that utilized prednisolone, lisinopril, warfarin, and dipyridamole	Relapse	Pfizer, 2nd dose	Not specified	Not specified.	Not specified. Mild proteinuria described, resolved after 3 days	Day 1: Macroscopic haematuria, resolved after 3 days	Not performed	Not specified	Not specified
Udagawa et al. [22], Japan	16	F	1.5 days	IgA nephropathy. Following multiple immunosuppressive treatments, including prednisolone, mizoribin, warfarin, and dipyridamole, patient was in remission.	Relapse	Pfizer, 2nd dose	Not specified	Not specified	15 months pre-vaccination: 0.5–1.0 No excess proteinuria described during acute episode	15 months pre-vaccination: Intermittent macroscopic haematuria Day 3: Macroscopic haematuria, resolved by Day 6	Not performed	Supportive treatment	Spontaneously resolved by 5 days following presentation. Serum creatinine did not increase and urinalysis has normalized
Abdel-Qader et al. [23] Jordan	12	M	Within 1 day	Nil	New-onset	Pfizer, 1st dose	Day 2: 1.77 Day 3: 1.74 Day 4: 1.59 Day 5: 1.39 Day 7: 1.39 4 days post-discharge: 0.87	Day 2: 4.1	Day 2: 1.7 Day 7: Trace protein 4 days post-discharge: Trace protein	Day 2: 1920 RBC/µL Day 7: 254 RBC/µL 4 days post-discharge: 992 RBC/µL	Mild increase of mesangial cells and matrix; no thickening of capillary loops, segmental sclerosis, crescent formation or necrosis were seen. Mild interstitial oedema was noted. Many of the tubules showed red cell casts with mild tubular injury and flattening of epithelial cells. Immunofluorescence showed granular mesangial deposits of IgA and C3.	IV pulsed methylprednisolone therapy was commenced.	Follow-up showed improved serum creatinine levels. Patient discharged on oral enalapril 5 mg, prednisolone 5 mg and esomeprazole 20 mg once daily

C3: Complement 3; F: Female; HPF: High-powered field; IgA: Immunoglobulin A; IV: Intravenous; M: Male; mg: milligram; RBC: Red Blood Cells; T1DM: Type 1 Diabetes Mellitus.

**Table 2:** Demographics and outcomes of children and adolescents with new-onset and relapsed nephrotic syndrome and tubulointerstitial nephritis following COVID-19 vaccination.

Author(s) & Country of Report	Age (yrs)	Sex	Time to Presentation from Day of Vaccination (Days)	Comorbidities	New- Onset or Relapse	Vaccine Brand & Vaccine Dose	Serum Creatinine Since Day of Presentation (mg/dL)	Serum Albumin Since Day of Presentation (g/dL)	Urine Protein-to-Creatinine Ratio Since Day of Presentation (g/g)	Haematuria Since Day of Presentation	Kidney Biopsy	Treatment Received	Clinical Outcome
Nakazawa et al. [24], Japan	15	M	1 day	Nil	New-onset	Pfizer, 1st dose	Day 1: 0.64	Day 1: 1.6	Day 1: 7.71	Day 1: Urine sediment showed <1 RBC/HPF	Not performed	Initiated on oral prednisolone 60 mg daily from 21 days post-vaccination	Achieved complete remission on day 12 of treatment. Did not develop complications such as hypertension, AKI or thrombus formation
Pella et al. [25], Greece	18	M	11 days	Nil	New-onset	Pfizer, 1st dose	19 days pre-vaccination: 0.98 Day 5: 0.79	Day 5: 1.8 Day 12: 1.8 Day 32: 3.2 Day 53: 4.0 Day 85: 4.3	Day 5: 2.0 Day 12: 10.5 Day 13: 23.4 Day 32: 1.2 Day 53: 0.5 Day 85: 0.2	Day 12: Urine sediment showed 2–3 RBC/HPF	Light microscopy showed no significant glomerular or tubular abnormalities. Immunofluorescence revealed no positive staining.	Commenced on 150 mg of Irbesartan and 48 mg of methylprednisolone on day 19 since presentation (day 6 of hospitalization).	Discharged on day 25 since presentation (day 12 of hospitalization). Irbesartan was stopped at follow-up review one week after discharge. Patient achieved complete remission of nephrotic syndrome at day 85 since presentation (7 weeks post-discharge).
Alhosaini et al. [26], UAE	16	M	7 days	Not specified	New-onset	Pfizer, 2nd dose	Day 1: 0.85 Day 5: 0.85 Day 22: 0.76	Day 1: 1.7 Day 5: 2.7 Day 22: 3.7	Day 1: 10.3 Day 5: 2.92 Day 22: 0.06	Day 1: ‘Moderate’ blood identified on urinalysis	Light microscopy showed normal glomeruli. Immunofluorescence studies were all negative. Electron microscopy showed diffuse foot process effacements. None of the glomeruli had any segmental sclerosis	The patient was commenced on oral prednisone 60 mg daily along with furosemide and olmesartan.	Clinical signs of nephrotic syndrome settled after 1 week since presentation, and proteinuria alongside serum albumin began to show improvement
Jongvilaikasem et al. [27], Thailand	14	M	5 days	Nil	New-onset	Pfizer, 1st dose	Day 1: 2.0 Day 5: 9.0 Day 40: 0.53	Day 1: 2.0	Day 1: 9.0 Day 40: 0.9	Nil throughout course of presentation	Light microscopy revealed unremarkable glomeruli. There was negative immunofluorescence staining. Electron microscopy showed diffuse foot process effacement. Diffuse tubular injury and interstitial inflammatory cell infiltration were noted.	Patient received acute haemodialysis for 3 weeks as became anuric with peak serum creatinine of 9.0 mg/dL on day 5 since presentation (10 days post-vaccination). Three daily doses of IV pulsed methylprednisolone were administered, followed by oral prednisolone 60 mg daily.	The patient achieved partial remission on day 40 since presentation, after a 5-week treatment of corticosteroids
Güngör et al. [28], Turkey	17	F	19 days	Minimal change disease diagnosed at 1.5 years old, was treated with corticosteroids, levamisole, mycophenolate mofetil and enalapril. The patient discontinued treatment 3.5 years ago and was in remission for 4.5 years	Relapse	Moderna, 2nd dose	Day 1: 0.5	Day 1: 1.2	Day 1: 8.7	Not specified	Not specified	Not specified	Not specified
Güngör et al. [28], Turkey	17.5	F	12 days	Diagnosed with idiopathic nephrotic syndrome at 2.5 years old. Patient received corticosteroid treatment for 6 months and had not relapsed on follow-up	Relapse	Brand not noted, 2nd dose	Day 1: 0.48	Day 1: 2.3	Day 1: 4.1	Not specified	Not performed	Patient commenced on oral corticosteroids. At time of case report, the patient is at a steroid reduction phase in which steroid treatment is intended to discontinue at the end of 6 months	Patient achieved remission 2 weeks following commencement of oral corticosteroids
Choi et al. [29], Korea	17	M	6 days	Nil	New-onset	Pfizer, 2nd dose	Day 3: 3.0 Day 5: 3.10 Please refer to Figure 3 of this referenced article by Choi et al. for the trend of serum creatinine changes throughout the course of presentation. Exact values not specified in the text by the authors.	Day 5: 0.1	Day 5: 4.3	Nil throughout course of presentation	Light microscopy showed glomerulus appearing slightly larger and segmentally hypercellular, involving mesangial cells. Interstitial infiltrates were mainly mononuclear. Focal and moderate interstitial fibrosis and tubular atrophy were noted in approximately 20% of the renal cortices. GBM showed focal wrinkling with partly irregular inner contours. There was negative immunofluorescence staining. Electron microscopy revealed non-significant focal epithelial foot process effacement with no electron-dense deposits.	Supportive treatment	Kidney function gradually improved, with the patient having increased oral intake. He was discharged after 1 week of hospitalization.
Choi et al. [29], Korea	12	M	18 days	Nil	New-onset	Pfizer, 2nd dose	Day 1: 2.28 Day 6: 2.25 Please refer to Figure 3 of this referenced article by Choi et al. for the trend of serum creatinine changes throughout the course of presentation. Exact values not specified in the text by the authors.	Day 6: 0.86	Day 6: 4.4	Day 1: 1+ blood on urinalysis	Tubules revealed severe necrosis, tubulorrhexis, and loss, with heavy infiltration of neutrophils, eosinophils, and mononuclear cells in the interstitium. GBM thickness was normal with smooth contours. There was negative immunofluorescence staining. No electron dense deposits observed	Commenced on oral steroids on day 10 of hospitalization	The patient had marked improvement in kidney function following commencement of oral steroids.

AKI: Acute Kidney Injury; COVID-19: Coronavirus Disease 2019; F: Female; GBM: Glomerular Basement Membrane; HPF: High-powered field; IV: Intravenous; M: Male; mg: milligram; RBC: Red Blood Cell.

## Data Availability

Not applicable.
